# Evolving funding strategies for research software: Insights from an international survey of research funders

**DOI:** 10.1371/journal.pone.0329833

**Published:** 2025-08-21

**Authors:** Eric A. Jensen, Daniel S. Katz

**Affiliations:** 1 University of Illinois at Urbana-Champaign, Urbana, Illinois, United States of America; 2 Institute for Methods Innovation, Casper, Wyoming, United States of America; CONRAD | Eastern Virginia Medical School (EVMS), UNITED STATES OF AMERICA

## Abstract

Contemporary research heavily depends on software. Research software, comprising source code, algorithms, scripts, computational workflows, and executables produced during or specifically for research, is crucial in advancing scholarly knowledge. However, this aspect of contemporary research can only thrive if research funders effectively support it. This survey study of international research funders addresses the research questions: 1) How do international funders currently support research software? 2) What challenges are funders aiming to tackle with their research software programs? 3) How successful do funders think their programs are? Survey results reveal a variegated funding landscape for research software, encompassing open-source projects, open-science tools, discipline-specific add-ons, infrastructure software, data science and AI tools, and general technology projects that include research software. Funders reported working to integrate research software into their formal definitions of research, codified in funding models and policies. Funders have been working to revise policies and adopt international frameworks such as ADORE.software to acknowledge research software’s role better. Respondents described innovative funding models designed to support research software more effectively than traditional research funding mechanisms. Supporting Research Software Engineers (RSEs) was another priority. Funding programs aimed to provide financial support, career development, and recognition for RSEs. Fostering collaboration between RSEs and other researchers was a less prevalent but noteworthy target for research funders. Promoting open-science principles and open source software development and maintenance was prioritized by research funders with targeted policies and programs. Overall, the reported initiatives aimed to ensure long-term research software accessibility, sustainability, and impact, with robust community engagement helping to contribute to a more effective research ecosystem. Finally, where funding programs for research software have been running for long enough to make an assessment, these efforts were overwhelmingly viewed as successful by the research funder representatives in our study.

## Introduction

Contemporary research heavily depends on software [1–2]. Research software—comprising source code, algorithms, scripts, computational workflows, and executables produced during or specifically for research—is increasingly essential in many academic disciplines [3–5]. However, the research software field faces numerous challenges undermining sustainability and impact (e.g., [6, 7]). Acknowledging this, some research funders have established programs and policies to extend research software’s impact [8–9].

No empirical study has examined how research software is supported across the highly variegated international funding landscape or the main challenges these funders aim to solve. Understanding these aspects can help pinpoint where current funding is focused and identify significant gaps. This manuscript addresses the following research questions: 1) How do international funders support research software? 2) What challenges are funders aiming to tackle with their research software programs? To gather answers to these questions, we surveyed research funders about the programs and policies they employ to bolster research software.

### Addressing critical challenges with research software funding

Over the past several decades, research software has progressed from a peripheral concern to a core focus of research funding. In the early decades of scientific computing, software development was typically supported indirectly as part of broader research grants or investments in computing infrastructure. Its development and maintenance were often treated as a secondary byproduct of research projects rather than a primary objective. However, as computational methods became central to more and more research fields, funding agencies gradually developed programs to provide dedicated support. This included funding initiatives by the National Science Foundation in the United States, establishing the Software Infrastructure for Sustained Innovation program, and the Software Sustainability Institute in the United Kingdom. These and other early initiatives marked a historical turning point, moving from ad hoc support toward structured, dedicated research software funding mechanisms.

Research software funding originates from government and philanthropic sources [10]. It aims to improve research efficiency, precision, and effectiveness, with wide-ranging benefits for science and society [11]. Funders seek to overcome critical challenges limiting research software’s impact, many of which center on the need to support those working in the research software field better, including research software engineers (RSEs) [12–13].

Recognition of research software contributions in hiring, promotion, and academic tenure remains insufficient [6]. Enhancing career pathways for individuals who develop and maintain research software is vital for their support [14–15]. A recent article offered ten simple rules to address this issue, emphasizing recognizing software contributions as valuable scholarly outputs [16] in line with open science principles [17]. Although these recommendations primarily target research institutions [18], research funders can influence the priorities and actions of these institutions.

Other known challenges include software skill gaps among researchers and research institutions [19–20]. Another gap is the need for funding to support the non-technical aspects of research software, including user experience design and community management [21–22].

Ensuring research software sustainability is another persistent challenge that has gained attention from research funders concerned about their investments in software delivering long-term benefits and not falling out of use prematurely [23]. This is a complicated, multi-faceted challenge [24–25], which is high on their agendas because it is central to research software’s ability to deliver lasting impact. Some funders address this challenge by promoting open-source software. While open source is emphasized in the research software community, it is far from universal.

In sum, research software funding has become a priority because software underpins scientific innovation, and investing in it yields broad benefits for efficiency, collaboration, and knowledge dissemination in the research community. In this study, international research funders are asked about the problems they aim to solve through their research software support and the specific programs and policies they have created to achieve this.

## Methods

An online mixed-methods survey was employed to examine the perspectives of research software funders. At the beginning of the self-administered survey, all participants gave written, electronic informed consent after reading through detailed consent text and having access to an information sheet. The University of Illinois Urbana-Champaign Institutional Review Board reviewed the study, provided consent text that was used and approved the study (no. 24374).

Data were gathered from December 2023 to May 2024, with participants spending an average of 28 minutes and 13 seconds to complete the survey. The data were cleaned by removing identifiable respondent details before analysis [26]. Participants provided explicit, written informed consent by clicking to indicate their approval at the start of the survey for research use and publication as open data post-anonymization. We deidentified the quotations in this article to comply with the terms of the research consent obtained.

### Survey design

The survey began by collecting profile information, including institutional affiliation and job title. The survey gathered detailed information about funders’ programs and policies to support research software. It also included a much smaller set of questions about additional topics, such as strategic funding priorities and awareness of key concepts. The data generated from this survey were too extensive to report in a single manuscript. Here, we focus on survey questions about how funders support research software and what problems they are targeting, as well as their assessments of the success of their programs. The data for this manuscript was gathered through the survey items shown in the Table 1 below.

**Table 1:** Details of survey structure.

Variable	Survey Item	Response Options
Policies, initiatives, or programs aimed at supporting research software	“Has your organization established any policies, initiatives or programs aimed at supporting research software?” (This could include grants, fellowships, funding policies, conference funding, or other kinds of support aimed at bolstering the sustainability or impact of research software)	Yes, No, Unsure (If ‘Yes’, then the next question was asked)
Number of policies or programs to be reported	“How many of your organization’s policies, initiatives or programs to support research software are you familiar with?”	1, 2, 3, 4, 5+
*The following questions were asked for each policy, initiative, or program*		
Name of policy or program	“Please name the policy, initiative or program (starting with the one you are most familiar with):”	[Text line]
Status of policy or program	“What is the status of this policy, initiative or program?”	Completed/closed, In progress/open, Other (please specify)
Link(s)/description	“Please provide link(s) to the policy, initiative or program, upload or email to [the researcher’s contact details].” “Link(s)/Description:” (If there is no documentation available, please describe it here:)	[Textarea], [File upload]
Type of policy or program	“Which of the following best describes the policy, initiative or program you named above?”	Funding program, Policy that affects funding decision-making or outcomes (funder side), Policy that affects funding applicants or recipients (applicant/awardee side), Other (please specify)
*If ‘Funding program’ was selected in the previous question, then the next question was asked*		
Type of funding	“Which of the following best describes the available funding?”	Funding that **includes** research software, Dedicated funding **only** for research software, Other (please specify)
*For all categories of policy, initiative or program, the following questions were asked.*		
Problem(s) addressed	“Please summarize the problem(s) this policy, initiative or program is aiming to address from your organization’s perspective:”	[Text Area]
Perceived level of program success	“What factors have contributed to its success or lack of success?”	Very successful, Successful, Neutral, Unsuccessful, Very unsuccessful, Not applicable/ No opinion

For quantitative survey results where multiple responses were allowed, *f* was used to signify the frequency of responses, as distinct from the number of cases (*n*).

### Sampling

Purposive sampling was adopted in this survey to strategically select a subset of international research funders deeply engaged in supporting research software initiatives. This sampling technique involved the researchers using their judgment to identify and include funders who could provide the most informative and relevant insights into funding practices, policies, and challenges specific to research software. While this approach enabled a focused exploration of critical issues within a specialized group, it also brought methodological limitations. Chief among these is the risk of selection bias, as the sample likely overrepresents the viewpoints of those who are already engaged on the topic of supporting research software while underrepresenting research funders who are not interested in research software. Additionally, because the sample is not randomly selected, the findings may not be generalizable to all international research funders, which could limit the broader applicability of the study’s results.

The survey focused on international research funders, including both governmental and non-governmental organizations such as philanthropic organizations. An initial contact list was developed based on participation in the Research Software Alliance (ReSA) and through the authors’ professional networks, explicitly targeting individuals known for their responsibilities in research software funding. This list was refined by removing those who had transitioned to unrelated professional roles or were unavailable for extended periods due to personal reasons.

**Fig 1:**
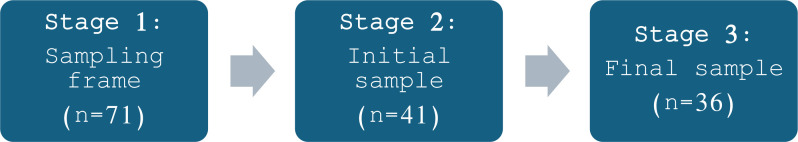
Sampling flow chart.

The sampling process was developed in stages (Fig 1). First, a contact list of 71 individuals from 37 funding organizations was compiled. Second, this list was refined by excluding people whose organizations had already provided a complete response or who were no longer engaged in relevant topics or were otherwise unavailable. This resulted in 41 individuals from 30 research funding organizations remaining in the sampling frame at the end of stage 2. Third, these 41 individuals were invited, however, five did not complete the survey. This resulted in a final sample of 36 participating individuals (representing 30 research funding organizations). The ratio between stage 2 and stage 3 equates to a response rate of 87.8% at the level of individuals.

Fully completed survey responses were not mandatory for inclusion in the sample. Because partially completed surveys were retained in the study, there are varied sample sizes across different survey questions. In addition, some questions were ‘check all that apply,’ allowing some respondents to select more than one category.

The individual respondents represented governmental (n = 28), philanthropic (n = 7), and corporate (n = 1) research funding organizations. Their job titles included roles in senior leadership and executive (e.g., Vice President of Strategy), program and project management (e.g., Senior Program Manager), planning and business development, and scientific, technical, and information technology (e.g., Scientific Information Lead).

Most survey participants were from North America and Europe, with 15 and 12 individual respondents, respectively. The sample also included four respondents from South America, three from Oceania, and one from Asia, indicating a global but uneven representation across continents. Some participating funders covered a wide range of disciplines, while others specialized in specific fields such as social sciences, health, environment, physical sciences, or humanities.

### Qualitative data analysis

A standard thematic qualitative analysis approach was employed with the open-ended results presented in this manuscript [27]. The process began by identifying themes and organizing the data accordingly. Relevant dimensions within each theme were also identified. Data extracts associated with each theme and its dimensions were then selected from the survey responses. Consistent with qualitative research methodology, the focus was on the presence or absence of ideas in the survey responses rather than quantification. However, prevalence was used to organize the results section, as more prevalent themes provided a more extensive set of data extracts and potential dimensions. The results section presents each theme in turn, with data extracts serving as evidence to support the descriptions of the themes.

## Results

The survey data sheds light on how funders are targeting their programs and policies to benefit research software. We begin with an overview of the quantitative results before digging into the details of the funding programs and policies research funders are deploying to support research software and its role in the wider research ecosystem. Key themes that emerged from open-ended survey responses are summarized in Table 2.

**Table 2:** Key themes from survey responses.

Key theme from qualitative results	Brief description
Integrating research software into funder definitions of research	Expanding existing open-science or data policies so software is formally treated as a core research output
Adapting traditional funding mechanisms for research software	Moving beyond short-term, novelty-focused grants toward models that fund long-term maintenance and community-driven projects
Funding and recognition for research software engineers (RSEs)	Creating dedicated salary lines, fellowships and career pathways that professionalise the RSE role
Fostering collaboration between RSEs and researchers	Supporting structured partnerships to improve code quality, scalability and uptake of modern computing within research
Promoting open science and open-source research software	Embedding open-source principles via targeted calls, policy revisions and capacity building to boost transparency and reuse

### Overview of funders’ research software support

Most respondents, 72.7% (n = 24), answered *yes* to the question, “Has your organization established any policies, initiatives, or programs aimed at supporting research software?”. 18.2% (n = 6) said *no*, and 9.1% (n = 3) were *unsure*. Those who answered *yes* were then asked for more specifics: Detailed reports were provided for an average (mean) of 2.83 funding programs and policies per respondent.

Respondents were asked to categorize these funding programs and policies into one of the following: *Funding program*, *policy that affects funding decision-making or outcomes (funder side)*, *policy that affects funding applicants or recipients (applicant/awardee side)* and *Other (please specify)*. *Funding programs* emerged as the most frequently reported (77%; f = 51). Policies that affect funding applicants or recipients on the applicant/awardee side were reported by 14% of respondents (f = 9). The least reported were policies that affect funding decision-making or outcomes on the funder side, accounting for 9% (f = 6).

Most of the reported funding programs were *open* (78%; f = 39) at the point that the survey response was completed, while 18% were *closed* (f = 10), and one reported program was created but not funded and launched yet (i.e., neither open nor closed). There were also four ‘other’ responses to this question, which included a focus on ‘guidelines’ for researchers supported by a particular research funder, national-level context and general principles for funding and support of research software and research software ‘policy [that] affects [the] funder and grantee side[s]’.

Among the respondents who provided information about their funding programs, 48% (n = 12) indicated that their organizations offer dedicated funding exclusively for research software. 52% (n = 13) reported that their organizations provide funding that includes research software as part of general research funding programs.

The survey responses revealed that the line between funding programs and policies was sometimes unclear. For example, a policy change may be implemented to expand an existing research funding program to include research software. In this case, the change could be viewed as a funding program for research software or a policy to support research software. For this reason, results are presented here, encompassing both funding programs and policies.

### Types of research software-related support

Funding categories included fellowships, grants for research software development and maintenance and other types of financial support. The main types and beneficiaries of research software funding are summarized in Table 3:

The list of funding support categories in Table 3 indicates the highly variegated nature of research software funding programs. However, open source was repeatedly mentioned as a focus for financial support.

**Table 3:** Types of research software-related funding.

Category	Funding Types
Open Source, Open Science, and Capacity	• Open-source software projects and communities • Open science software tools, methods, and capacity building
Training	• Professional development for researchers and students to train them in leveraging research software
Add-On for Research Projects	• Research software or digital infrastructure for particular disciplines as add-on mini-projects within a larger research project
Innovative or Computationally Challenging Methods	• Research and refinement of experimental, innovative, or computationally complex methods and techniques • Data science software • AI tools
Sustaining Existing Software	• Sustaining existing research software
Technology Project Funding	• Research software projects with no specific topic limitations • General technology project support, including research software
Core Research Infrastructure	• Software that forms part of the infrastructure for research
External Software Services and Support	• IT professionals to join research projects • Hiring companies to help produce research software for research projects

### Challenges addressed by funders’ programs and policies

The survey asked respondents to describe the specific problems their policy, initiative, or program aimed to address from their organizations’ perspective. Responses revealed how research funders are evolving their programs and policies to suit research software’s needs more effectively.

#### Integrating ‘research software’ within funder definitions of ‘research’.

Respondents expressed a growing recognition of the importance of incorporating research software into existing funding policies. One funder representative described efforts to integrate research software into existing policies:

“We have an open access policy and a research data management policy. As a funder […] committed to open science in its broadest sense, we now want to expand [our] research data management policy to include a broader range of outputs, including research software. […] We are in the process of revising and updating […] our current data management policy. [The national research funder] is also about to sign the Amsterdam Declaration on Funding Research Software Sustainability [ADORE.software]. One of the recommendations in the Declaration is to include research software in open-science policies, which we plan to do by broadening the scope of our research data management policy.”

This response indicates a reorientation from historically concentrating on open access and research data management to beginning to treat software as a similarly vital research output. This also points to the role of international collaboration between research funders in informing policy transformations to accommodate research software.

Another response also made the influence of such international engagement between funders explicit:

“Becoming [ADORE.software] signatories will provide us with an international framework that will inform our policies, initiatives, and programs around research software sustainability.”

Another funder representative highlighted an effort to address the lack of recognition of research software in academic career progression and assessment:

“Recognition: Research software is not yet recognized as a research product equally important as publications or, to a lesser extent, datasets. The lack of recognition in career progression or assessment of contributions by researchers related to research software is a major obstacle to advance in our policies.”

This response acknowledges that contributions to software development do not yet carry the same weight as publications or datasets in researchers’ professional evaluations. This gap makes it challenging to secure sustained support for software efforts.

Another respondent highlighted the need to amend the organization’s funding programs and policies to integrate research software:

“Identify possibilities to change/ evolve existing funding programs and existing policies to better include [research software] funding in established programs.”

This data extract points to the need to change existing modes of research funding to provide appropriate support for research software.

One respondent explained why such change may be slow in coming for some research funders, citing the complexity of research software as a research output as a barrier to progress in evolving research funding policies.

“Complexity [is a challenge for research software funding]: the intrinsic complexity of research software compared to other research digital objects (e.g., publications or datasets) requires more sophisticated tools and mechanisms to ensure its maintenance, re-usability, adequate metadata characterization etc.”

As this funder notes, compared to publications or datasets, software brings greater challenges for long-term sustainability. Accounting for this is one reason that simply extending existing research funding to encompass software may be an insufficient solution.

As the data extracts above show, fully integrating research software into the definition of ‘research’ is still a work in progress for international research funders. Indeed, one respondent noted that a strategy for research software “has been completed, but is only now being used to facilitate the specific programs that […] will launch in [the next several years]”, while another said a research funding program was “*currently being modified* to include research software” (emphasis added). Such responses show that research software funding is still in flux. Nevertheless, this broadening of funder mandates reflects a desire to keep pace with the sophistication of modern research outputs, where software is now foundational across many disciplines.

#### Adapting traditional funding mechanisms for research software.

The survey responses reflected widespread recognition of the necessity of changing funding models to go beyond traditional mechanisms focused solely on research projects. Traditional research funding mechanisms often center on short-term, novelty-driven projects, limiting their capacity to sustain and evolve software that underpins much of modern scientific inquiry. Survey responses point to an emerging consensus that research software requires a distinct approach to ensure stability, foster community collaboration, and address long-term maintenance.

Participants noted that existing funding structures are insufficient for supporting the long-term sustainability and community engagement necessary for research software. One respondent expressed concern that traditional funding structures overemphasize novelty in a way that is poorly aligned with research software.

“Current funding mechanisms in [country] (and elsewhere) tend to focus on enabling novel research. There is a need for funding mechanisms that instead favor a community-driven approach to software development and that promote a culture of building upon existing efforts [...] to build better and more broadly used research software.”

Another respondent highlighted the specific example of supporting community involvement in research software projects to exemplify the need for policy change:

“Community building is hard to support with traditional [research] funding mechanisms. This aims to help pay contributors and community members and ensure diversity, equity, and inclusion in open source.”

Some funders reported exploring innovative funding mechanisms as a concrete change to better support research software’s role:

“We are going to trial funding software via a different mechanism: via grant holders sub-awarding to software teams that maintain the software they use. We are hoping that this will lead to greater recognition of the need to maintain software for research, and in the long term regular funding to support highly used software.”

Another respondent discussed the introduction of programs that specifically fund research software maintenance:

“This program funded projects focused on software maintenance and development, not just ‘new’ projects.”

Another response cited infrastructure software as another example of unique research software funding needs that require changes to traditional funding models:

“Aims to support more infrastructural software that wouldn’t be supported by a disciplinary research funding program.”

In sum, many respondents noted that research software has unique funding requirements that traditional research funding models insufficiently address. This is the main rationale for an ongoing evolutionary process in research funding aimed at improving support for research software.

#### Funding for research software engineers (RSEs).

The survey data show a growing awareness among funders that research software engineers (RSEs) are crucial for advancing open science and sustaining software infrastructure. Traditional funding mechanisms have typically concentrated on short-term research projects led by principal investigators, leaving RSEs with limited pathways to long-term support and career development. New initiatives seek to address these shortcomings by offering dedicated salary support, professional recognition, and autonomy for RSEs.

Indeed, respondents underscored the importance of funding personnel who develop and maintain research software, recognizing that new programs and policies were needed to support RSEs. An example of a funding program directly supporting RSEs was described as follows:

“‘The [specific funding program] would support research software engineers building sustainable open software and tools [...] providing salary support and autonomy.”

Support for professional growth among RSEs and other “digital professionals” in research was also noted:

“Supporting RSEs and digital professionals to develop career paths and share knowledge.”

Another reported the introduction of a funding program aimed at addressing the unmet need for software engineers in research projects:

“Lack of proper support of software engineers to produce high-quality software for the research projects. [My organization’s funding] program offers a means to hire software engineers. […] With this policy, [a proportion] of a given research grant can be used to hire a small or medium software company to help produce/package/maintain the software.”

This policy was aimed at addressing a perceived “lack of proper personnel in a research institution to produce high-quality software.”

Funders reported introducing programs that provided both financial support and recognition to individuals leading in developing research software. These programs aimed to acknowledge the contributions of RSEs and offered them resources to continue their work long-term. One program was created to support RSEs who may not hold principal investigator roles within a research institution:

“The [funding program] would support research software engineers building sustainable open software and tools. It will use a mechanism designed to support stable research and career opportunities for researchers in an existing research program who may not serve as principal investigators (PIs) by providing salary support and autonomy. The award is 2 years or less, and the budget will support the research software engineer’s salary, commensurate with the existing program, for at least 6 months per year.”

Such responses indicated an evolving effort by some funders to support RSEs in developing their careers and networks. A participant described multiple initiatives in this area:

“Supporting RSEs and digital professionals to develop career paths and share knowledge. [Our specific program] is aimed at supporting software sustainability. The [program] aims to recognize the contribution of RSEs who are driving the development of high-quality research software and demonstrating leadership in embedding the vital role of software in disciplinary and institutional research cultures. [My research funding organization] aims to fund projects where the proposal provides evidence of an established or growing demand for the proposed software to facilitate more effective or efficient research workflows.”

While the respondents highlighted programs they were introducing to support RSEs, one respondent noted the persistent challenges faced by emerging RSE communities, especially in regions with limited financial and other resources (e.g., Kozma et al., 2018):

“Challenges/problems to address: RSE as a nascent field in [in a low/middle income world region], limited training and support, limited infrastructure, limited and unsustainable funding.”

Ultimately, respondents viewed RSEs as an essential component of a more effective and sustainable research software ecosystem. Creating such programs required adjustments to longstanding research funding programs and policies.

#### Fostering collaboration between RSEs and other academic researchers.

A less prevalent but still notable theme among research software funders was supporting collaboration between RSEs and other academic researchers. Such programs offer structured support to improve code quality, scalability, and adoption of cutting-edge computing technologies.

Funders have begun establishing programs specifically designed to facilitate these collaborations, as can be seen in the following example:

“Support collaborations between scientists [in a particular academic discipline] and software engineers to enhance the design, implementation, and ‘cloud-readiness’ of research software.”

Such programs indicate a developing commitment to bringing together interdisciplinary expertise to improve research software capabilities. The data extract below recognizes the need for interdisciplinary teams, including research software expertise, to foster innovation.

“Scientific and engineering innovations by interdisciplinary teams develop novel methods to collect, sense, connect, analyze, and interpret data from individuals, devices, and systems, enabling discovery and optimizing [positive societal outcomes].”

Funder representatives in our survey also focused on enabling links between researchers and software developers to leverage modern computing technologies within the research enterprise.

“Create vibrant partnerships with creators and developers of software and tools to leverage modern computing in the research enterprise.”

Overall, these efforts aimed to enhance research capabilities by fostering cooperation between scientists and software professionals.

#### Promoting open science and open-source research software.

Research software funders prioritized funding programs and policies to support open-source research software and open science principles to increase reuse. Survey responses reveal multiple initiatives designed to embed open practices across the research lifecycle, anchored by updated policies and dedicated funding programs. These moves underscore the belief that open-source software enhances transparency, reproducibility, and community engagement in science.

For example, one funder representative underscored that open science is a priority that affects the organization’s support for research software:

“Open science is a core value of [my research funding organization], and our policies for grantees attempt to support this. There are additional [open science] requirements for our […] grantees [affecting project delivery] as well.”

Additionally, policies were crafted to address the sharing of software

“The policy addresses the requirements around sharing of scientific software developed by the science mission directorate. It does touch on requirements for a number of different organizations but it primarily focused on those producing the information.”

Several funders established programs specifically to support the maintenance and development of open-source research software. One example is detailed below:

“[We have] a funding program that supports software maintenance, growth, development, and community engagement for critical open source tools.”

Another respondent detailed a program introduced to enhance research by supporting associated research software:

“Whether it’s hiring an additional developer, improving documentation, addressing usability, improving compatibility, onboarding contributors, or convening a community, the program aims to help make the computational foundations of research [within a particular academic discipline] more usable and robust.”

Programs were implemented to provide sustainable funding to open-source software, as a supporting or ‘enhancing’ aspect of the primary funding for other kinds of research. Examples of this can be seen in the two data extracts below:

“The program provides sustainable funding to open source software that supports the science mission.”

“The program supports the modernization and release as open source of research software. This is an enhancement to existing grants. This program was expanded to include additional open science considerations [recently].”

In addition, programs aimed at supporting open-source research software also included capacity building:

“[We fund] a training program that includes a curriculum on the open sharing of scientific processes and products, including code. Sharing of code is still something relatively new for many community members even though there are many experiences, tools, and practices that are available to make it easier.”

These data extracts exemplify the strong emphasis on supporting open source among research funders. They show a concerted effort among funders to prioritize the openness of research software through targeted funding calls, policy revisions, and supportive infrastructure.

### Funders’ assessments of program success

Funders generally perceived their research software programs and policies as successful, with the majority characterizing them as either “very successful” or “successful.” Explanations for these high satisfaction levels centered on flexibility in program design, responsiveness to community needs, and evolving internal processes within funding organizations.

After reporting the programs and policies their organizations have developed to support research software, respondents answered a question (f = 55) about their level of success: “How successful has this policy, initiative or program been to date?”. After removing the ‘not applicable/ no opinion’ responses (f = 12), the remaining results are presented in Table 4.

**Table 4:** Perceived success of programs and policies to support research software.

Perceived program success	Responses	Percentage
*Very successful*	19	44.1
*Successful*	18	41.9
*Neutral*	6	14
*Unsuccessful*	0	0
*Very unsuccessful*	0	0
Total	**43**	**100**

The set of responses shown in Table 4 is skewed towards the view that these programs are successful, with the rest of the responses being ‘neutral’. A follow-up question probed for explanations of these assessments of success. Responses clarified what research funders viewed as drivers of success for these programs and policies.

### Research funders’ explanations of success drivers

Research funders identified several key factors that they believe contributed to the averred success of their research software funding programs and policies.

#### Flexibility and iterative development.

Funders emphasized the importance of customizing their programs to the diverse needs of the research software community. One respondent expressed this as follows:

“Flexibility, agility.” (Very successful)

One respondent indicated that programs were more effective when focused on research software per se:

“Funds can be used for any needs associated with research software, rather than research.” (Very successful)

Another respondent pointed to the value of flexibility in grant administration in terms of eligibility and funding timeframes.

“Ability to provide flexibility in funding (e.g., funding internationally, and allowing no cost extensions).” (Very successful)

Such adaptable funding mechanisms allowed funding programs to respond to variegated and emerging research software needs.

“[A driver of success was offering a] very capacious program that can fund research at numerous stages, from early development to sustaining mature [research software]. Growth in the [field of scholarly research targeted by the program].” (Very successful)

This focus on the distinctive needs of research software was noted as a key driver of success.

In addition, respondents pointed to the process of adapting the research software funding programs based on research and feedback.

“Careful analysis of the issues, multiple feedback rounds, excellent intellectual input […], supporting studies.” (Very successful)

One respondent reported centering ‘learning’ and development in the research software funding design as important for success:

“Started small—only open to granted projects, enthusiastic reviewers, possibility for open and detailed discussion with reviewers, furthering our development of this funding initiative, we are a learning organization.” (Successful)

Overall, research funders saw the benefits of taking an agile, developmental approach to supporting research software.

Funders also aimed to show agility by offering long-term funding for sustainability and maintenance rather than limiting support to novel research software

“The program focused on funding sustainability rather than innovative work that helps support the maintenance of the programs. For a number of projects, it also helped grow a more diverse network of maintainers and community members.” (Very successful)

As shown in the data extract above, the perceived success of these funding programs was also linked to the research software community and the wider research community.

#### Research community engagement.

Building on demand from the research community was also noted as a success driver.

“It’s bottom-up initiative and scientists like it very much.” (Very successful)

Respondents indicated that the research community’s input was key to delivering a successful funding program.

“The desire in the community for change has been a strong positive force.” (Successful)

Indeed, programs that garnered heavy interest from researchers were viewed as successful:

“The program was well subscribed by investigators and a number of publications and new software code was developed.” (Very successful)

“Success: many thousands of these fellowships have been granted in the past 20 years.” (Successful)

Some programs faced challenges in gaining applications, which caused some hesitation about judgements of ‘success’.

“The initial years of the program attracted limited interest, and it is still early to judge how those programs have worked on converting and releasing their software.” (Successful)

While strong community interest was generally viewed as a marker of success, one respondent highlighted that it sometimes led to challenges:

“Strong need in the community for these mechanisms. One issue was that the call was incredibly oversubscribed, leading to issues in the review process and a feeling of ‘stochasticity’ among reviewers and panelists.” (Successful)

These data extracts reveal factors that research funders consider when assessing a program’s success.

#### Internal processes within research funders.

Processes within the research funding organizations represented by our respondents were highlighted as affecting the success of research software funding. For example, having specialized program leads involved in reviewing all kinds of research proposals was viewed as enabling more effective research software funding.

“Successful because all program [leads] read all proposals [that our organization receives], which means that the [program lead] responsible for [software] (and thus most focused on this information) can read and comment on all proposals.” (Successful)

One respondent noted that their requirements in research software funding programs were not consistently enforced, with the organization instead focusing on developing the capabilities of grantees.

“We aren’t always as thorough as possible in enforcing our requirements, especially for grantees in other [research] programs. We also continue dedicating additional resources (e.g., for training) to help our grantees meet these expectations.” (Successful)

These results offer a window into some of the internal processes that may be affecting research software funding success.

### Explaining ‘neutral’ and ‘not applicable/ no opinion’ responses regarding program success

Because none of our respondents reported an ‘unsuccessful’ or ‘very unsuccessful’ program, the only remaining categories were ‘neutral’ and ‘not applicable/ no opinion’.

#### Newness of programs and policies.

Many respondents indicated their initiatives were not mature enough to evaluate their success effectively.

“The program has just been released!” (Neutral)

“The grants are only just starting.” (Not applicable/ no opinion)

“Project is in the early stages, so not yet sure what the impact will be.” (Not applicable/ no opinion)

“Work still underway, and has been slow to commence due to contracting and resourcing challenges.” (Neutral)

“Program still getting going.” (Neutral)

For these respondents, the unfolding nature of these programs forestalled any assessment of success or failure.

#### Preliminary signs of influence.

Despite still being at an early stage of implementation, one respondent observed that their new research software policy had begun to affect other policies indirectly:

“It is still too early to assess its outcomes of the policy. Indirectly, it has been somewhat successful in influencing other policies.” (Not applicable/ no opinion)

This suggests that even without direct, measurable outcomes, some funders may be prepared to infer success from influence on organizational policies.

## Discussion

Research funders often face competing pressures from top-down government and sponsor priorities [28] and bottom-up criticisms for shortfalls in addressing the research community’s needs [29–31]. Here, we focus on the specific challenge of transforming longstanding research funding models to address the evolving needs of research software and the wider research ecosystem [32–33].

Even though the survey did not set out to measure changes in policies and programs, the open-ended responses nevertheless made clear that such changes are underway. By implementing new funding programs, providing supplemental funding, amending policies and experimenting with alternative funding models, research funders aimed to ensure the longevity and robustness of essential research software tools. Where programs have been running for long enough to make an assessment, these efforts were overwhelmingly viewed as successful by the research funder representatives in our study. Taking a flexible, community-informed, and internally coordinated approach to research software funding was identified as key to this perceived success.

Overall, survey results reveal an evolving process of reforming funding programs so that maintaining, refining, and enhancing research software becomes a routine and adequately supported research activity. Funders reported exploring innovative funding approaches to offer greater flexibility and responsiveness. Traditional funding models were viewed as failing to support the long-term maintenance, community building, and infrastructural needs crucial for the sustainability of research software. Respondents reported on dedicated funding programs designed to address these challenges and adjusted existing policies to value research software as a critical research output.

Research funders evinced a growing acknowledgment of the essential contributions of research software engineers (RSEs). Respondents showed a commitment to integrating RSEs into the research ecosystem. Funders are implementing initiatives such as funding and fellowship programs that provide financial resources and recognition for RSEs. Career development opportunities are being created to help RSEs advance professionally and share knowledge within the community. These efforts aim to enhance the professional development of RSEs and integrate them more fully into the broader research community [34]. Mechanisms for hiring RSEs or external companies were reported, seeking to help research institutions lacking sufficient in-house expertise to produce high-quality software. The survey responses reflect a concerted effort by research software funders to enhance collaboration between scientists and research software engineers. By promoting interdisciplinary partnerships, building inclusive communities, and implementing supportive policies, funders aim to leverage the combined expertise of both scientists and software engineers. Creating targeted programs that support professional growth, allocate dedicated resources, and endorse RSE leadership can offer a more durable path for software development and integration in research, particularly as the global demand for robust and reusable research software continues to rise.

International research funders participating in our study recognized the unique funding needs of research software. By addressing the limitations of existing research funding programs and policies, funders are working to foster a sustainable and collaborative research software ecosystem within scholarly research. The survey results show that many research funders are facing the need to expand their support mechanisms to include long-term funding for research software development and maintenance. Understanding that software is foundational to contemporary research, funders are increasingly adopting flexible funding models that can adapt to the dynamic needs of software projects. Funders support a more collaborative and efficient research environment by investing in open-source projects and encouraging transparency and reusability. Funders seek to expand software innovations’ reach and impact by explicitly valuing open-source methods and fostering skills development, making them more accessible and sustainable across research domains. As more funders formally acknowledge software as a significant aspect of research, they will increasingly focus on ensuring their programs and policies deliver effective support for research software.

At the same time, it is important to remember that this study used a purposive sampling approach to gain insights from those actively involved in discussions about research software funding. This means that the responses we received likely comprise a ‘best-case scenario’ for research software. Undoubtedly, significant work is ahead to extend research software funding good practices to a broader set of research funders.

## Conclusion

The survey results illuminate an evolution in how research funders perceive and support research software, with notable implications for both funders and the research software field. Integrating research software into funders’ definitions of ‘research’ signifies a paradigm shift acknowledging software as a tool for others to use and as a fundamental research output. By embracing and implementing international policy declarations such as ADORE.software [35], funders are developing more comprehensive policies that support research software’s role.

Adapting traditional funding mechanisms to suit the unique needs of research software highlights a critical reevaluation of funding models [36], which can be difficult for research funding organizations. Understanding that traditional grant structures are incongruent with the iterative, long-term and community-oriented nature of research software development and maintenance is one thing. The move towards funding models that support community-driven development and research software sustainability is another, much more challenging level. Those international research funders taking on this challenge deserve praise for innovating to meet the needs of this critical aspect of contemporary research.

The emphasis we found on supporting RSEs addresses the human capital component essential for research software to thrive [19]. Through programs offering financial support and career development opportunities for RSEs, funders are investing in professionalizing roles that bridge the gap between software engineering and other research capacities. Investment in RSEs can be expected to enhance productivity and innovation within the research community. However, challenges remain particularly acute in resource-limited regions, indicating a need for global strategies to build capacity and equity in the research software workforce. Moreover, the ongoing development of funding programs fostering collaboration between RSEs and other academic researchers is also promising. The emergence of such funding programs reflects an understanding that scientific progress increasingly depends on robust software and that well-orchestrated partnerships can amplify both discovery and societal impact. Enabling diverse connections like this within the research ecosystem can facilitate the flow of knowledge and accelerate innovation.

The promotion of open science and open-source research software by funders also has important implications [37]. Open-source software fosters an environment of transparency and communal contribution, which is essential for a healthy research ecosystem [8,38,39]. These values also align with the principles of the open science movement [40].

In conclusion, the survey results suggest that some international research funders increasingly acknowledge the multifaceted value of research software, leading to policy and programmatic changes supporting its development, sustainability, and integration into the broader research ecosystem [41]. For the research software field, these changes present opportunities for greater recognition [39], sustainable funding, inclusion [42] and enhanced collaboration, which could drive innovation, ethical impact [43] and efficiency in scholarly research. The key will be for good practices in research software funding to be continually improved and applied on a broader scale.
